# Targeted Magnetic Nanoparticles for Beta-Amyloid Detection

**DOI:** 10.3390/pharmaceutics16111395

**Published:** 2024-10-29

**Authors:** Nelly S. Chmelyuk, Aleksey A. Nikitin, Veronika V. Vadekhina, Vladimir A. Mitkevich, Maxim A. Abakumov

**Affiliations:** 1 Department of Medical Nanobiotechnology, Pirogov Russian National Research Medical University, Ostrovitianov Street, 1, 117997 Moscow, Russia; nellichmelyuk@yandex.ru (N.S.C.); nikitin.chemistry@mail.ru (A.A.N.); veronikavadehina@gmail.com (V.V.V.); 2 Laboratory of Biomedical Nanomaterials, National Research Technological University “MISIS”, Leninsky Prospekt, 4, 119049 Moscow, Russia; 3 Department of Basic and Applied Neurobiology, Serbsky National Medical Research Center for Psychiatry and Narcology, 119991 Moscow, Russia; 4 Engelhardt Institute of Molecular Biology, Vavilov Street, 32, 119991 Moscow, Russia; mitkevich@gmail.com

**Keywords:** Alzheimer’s disease, HAEE, magnetic nanoparticles, amyloid fibrils

## Abstract

**Background/Objectivities:** The presence of beta-amyloid plaques is a part of the pathogenesis of Alzheimer’s disease, but there is currently no universally accepted method for magnetic resonance (MR) imaging of the disease. However, it is known that magnetic nanoparticles (MNPs) can improve the T2 contrast in MR images of various targets. **Methods**: We used cubic MNPs, which were produced by thermal decomposition and then it was covalently bonded to a modified fluorescently labeled tetrapeptide, HAEE-Cy5, for visualizing beta-amyloid plaques. The interaction of MNPs-HAEE-Cy5 and beta-amyloid was determinate by confocal microscopy using SH-SY5Y cell line. **Results:** MNPs exhibit relatively high relaxivity (approximately 200 mM^−1^s^−1^), which is crucial for enhancing target visibility in MR imaging. HAEE provides targeted delivery of MNPs by specifically interacting with beta-amyloid, while the fluorescent label Cy5 enables monitoring the efficacy of the interaction through confocal microscopy. **Conclusions:** The MNPs modified with HAEE-Cy5 demonstrated excellent binding to beta-amyloid plaques in vitro, as shown by experiments on the SH-SY5Y cell line. These results suggest that the proposed method has potential for use in future MR imaging studies of Alzheimer’s disease.

## 1. Introduction

Alzheimer’s disease (AD) is a progressive neurodegenerative disorder that presents challenges in therapy and instrumental diagnostics, including magnetic resonance imaging (MRI) [1]. Currently, only maintenance therapy is available for patients [2].

One of the main hallmarks of AD is the formation of senile plaques and fibrils in the brain, the major component of which is beta amyloid (Aβ). These plaques and fibrils are formed by the aggregation of Aβ peptide, derived from the amyloid precursor protein (APP), through its cleavage by β-secretase to β-CTF and then by γ-secretase to monomeric Aβ [3].

The tetrapeptide Ac-HAEE-NH_2_ has been introduced as an anti-amyloid agent capable of interrupting the formation of beta-amyloid aggregates [4]. Additionally, HAEE protects α4β2 nicotinic acetylcholine receptors (nAChR) from inhibition by Aβ [5]. The HAEE sequence is found in α4β2 nAChR and interacts with the 11EVHH14 sequence of Aβ. It was also shown that the addition of HAEE to cells preincubated with preformed Aβ fibrils reduces the inhibition of nAchRs, thus restoring the ability of cells to respond to nAchRs agonists. Taken together, these data allows us to propose that HAEE can efficiently bind with Aβ fibrils, potentially disrupting their formation [5].

For now, there is no specific MRI label or contrast agent allowing us to non-invasively detect Aβ fibrils in the brain for patients in clinical practice. One promising material providing strong MRI contrast is magnetic nanoparticles (MNPs) based on iron oxide. Recently, it was shown that conjugation of MNPs with targeting ligands enables specific visualization of different molecules, so-called “molecular imaging”. Currently, there are methods for imaging tumors, myocardial fibrosis, etc. [6]. Molecular imaging for AD is still in its early stages [7]. Some studies have used MNPs to visualize Aβ plaques in the brain, with targeting vectors including hyaluronic acid in one case [8] and a phenothiazine-based near-infrared fluorescent dye in another case [9]. However, for the realization of Aβ molecular imaging via MRI for AD diagnostics, targeting ligands specific to Aβ fibrils is needed.

In this work, we present the promising Aβ-specific MRI contrast agent that consists of the tetrapeptide HAEE covalently conjugated with MNPs through a peptide linker. The modification of the MNPs by 3,4-dihydroxyphenylacetic acid (DOPAC) and carboxy-terminated polyethylene glycol (PEG) forms a thin layer, which contains functional carboxylic groups for subsequent conjugation with the HAEE derivative and allows to stabilize the MNPs in aqueous solutions. The introduction of the Cy5 label during the synthesis of HAEE makes it possible to detect the specific binding of the MNPs-HAEE-Cy5 to Aβ fibrils on SH-SY5Y cell surfaces by fluorescent methods, compared to MNPs labeled only with Cy5.

## 2. Materials and Methods

### 2.1. Materials

The following reagents were used to obtain, modify, and functionalize MNPs: oleic acid (C_12_H_34_O_2_, 90%, Sigma-Aldrich, St. Louis, MO, USA), oleylamine (C_18_H_35_NH_2_, 70%, Sigma-Aldrich), 1-octadecene (C_18_H_36_, 90%, Sigma Aldrich), tris(acetylacetonato) iron (III) (Fe(acac)3, 99.9%, Sigma Aldrich), acetone (C_3_H_6_O, Reakhim, Ekaterinburg, Russia), hexane (C_6_H_14_, Reakhim), 3,4-dihydroxyphenylacetic acid (DOPAC, Sigma-Aldrich, USA), sodium hydroxide (NaOH, 97%, Sigma-Aldrich, St. Louis, MO, USA), methyl alcohol (CH_3_OH, 95%, BioPharmCombinat, Moscow, Russia), N-hydroxysuccinimide (NHS, 98%, Sigma-Aldrich, USA), 1-ethyl-3-(3-dimethylaminopropyl)carbodiimide (EDC, 98%, Sigma-Aldrich, USA), poly(ethylene glycol) 2-aminoethyl ester of acetic acid (Mn 1.100, Sigma Aldrich, USA), Cy5-amine (lumiprobe, Moscow, Russia), chloroform (CH_3_Cl, Component-Reaktiv, Moscow, Russia), 3-(2-pyridyl)-5,6-diphenyl-1,2,4 monosodium salt hydrate -triazine-p,p′-disulfonic acid (ferrozine, C_20_H_13_N_4_NaO_6_S_2_xH_2_O, 97%, Sigma Aldrich), concentrated hydrochloric acid (HCl, 36%, Reakhim), Amicon Ultra 30 kDa centrifuge filters. For the preparation of all solutions in the processes of synthesis and analysis, we used deionized distilled (DI) water prepared in a Milli-Q-RO4 system (Millipore, Burlington, MA, USA). HAEE-Cy5 (synthesized by Syntol), for the preparation of sodium phosphate-buffer solution (1× PBS, Sigma-Aldrich), deionized distilled (DI) water, and Amicon Ultra 10 and 30 kDa centrifuge filters.

### 2.2. Methods

#### 2.2.1. Dye Labeling of Aβ_1–42_

For amyloid fibrils, modeling we chose synthetic peptides Aβ_40_ and Aβ_42_ from BioPeptide (San Diego, CA, USA), stored at −70 °C. The amyloid sample was prepared using standard technology [10]. On ice, 1 mg of the powder was dissolved in 1 mL of 1,1,1,3,3,3-hexafluoro-2-propanol to obtain a final peptide concentration of 1 mM. After dissolution, the solution was incubated for 1 h at room temperature to form peptide monomers. Then, the vial with the peptide was placed on ice for 5–10 min, and the resulting solution was transferred to microtubes. The microtubes were opened to evaporate 1,1,1,3,3,3-hexafluoro-2-propanol for 1 h on a rotary evaporator, and the remaining alcohol was removed. The resulting films were then stored at −70 °C. Then, 100 μg Aβ was dissolved in 100 μL PBS, and 100 μg NHS-AF-488 (20 mg/mL) was added to the peptide. The mixture was incubated at 4–6 °C for 60 min. Aβ-AF488 was washed ten times with PBS at 4–6 °C using centrifugal filters (Millipore Amicon Ultra-4, MWCO 3 kDa).

#### 2.2.2. ThT Assay

Aβ-films were dissolved in DMSO to 2.5 mg * mL^−1^, then 10 μM ThT and 10 μM Aβ were dissolved in PBS in 384-cell wells and incubated at 37 °C for 4 h. The intensity of fluorescence was measured at 490 nm (λex = 450 nm) using an EnSpire microplate reader (PerkinElmer, Waltham, MA, USA).

#### 2.2.3. Synthesis of MNPs

Synthesis of MNPs was carried out according to the previously described protocols with some modifications [10,11]. 0.5 mmol tris(acetylacetonato) iron (III) Fe(acac)**_3_**, 8 mmol oleic acid, 2 mmol oleylamine, 4 mmol 1,2-hexadecanediol and 10 mL dibenzyl ether were placed in a 100 mL three-necked round bottom flask, equipped with a reflux condenser and thermometer. First, the reaction mixture was heated up to 130 °C under argon flow and maintained for 30 min. Then, the mixture was heated up to 280 °C with a rate of 3 °C/min and maintained for another 2 or 4 h. After cooling the solution to room temperature, nanoparticles were separated from the solution by centrifugation for 30 min at 6000 rpm, after which the formed precipitate was redissolved in toluene.

#### 2.2.4. Characterization of Synthesized MNPs

Size and morphology were analyzed using a JEOL JEM-1400 microscope (JEOL, Tokyo, Japan) operated at a 120 kV acceleration voltage. Overview images were taken in conventional bright-field transmission mode. Samples were prepared by casting and evaporating a droplet of solution onto a carbon-coated copper grid (300 mesh). The average diameter of MNPs was calculated from TEM images by an analysis of about 500 NPs for each sample using ImageJ 13.0.6 software.

Measurements of static magnetic properties (from −1500 to 1500 kA/m, 300 K) were carried out using a Quantum Design PPPMS-9 (Quantum Design, San Diego, CA, USA with 2 mm amplitude of oscillations, 40 Hz frequency.

Structural phase analysis was studied on an X-ray diffractometer DRON-4 (LNPO “Burevestnik”, Saint Petersburg, Russia) with Co-Kα radiation (λ = 0.179 nm), tube current 19 mA, voltage 40 kV. The tube operated in standard mode. The survey was carried out at diffraction angles 2Θ from 20° to 120° with a scanning rate of 0.1° and an exposure time at the shooting point of 5 s. Qualitative phase analysis was carried out by comparing the spectra using PDXL 1.8 software: Integrated software for X-ray powder diffraction; the crystallized size and microstrains were calculated using line profile analysis in the Rietveld method and whole powder pattern fitting (WPPF Analysis).

The hydrodynamic diameter and polydispersity index (PDI) were measured by the dynamic light scattering (DLS) method using a Malvern Zetasizer Nano ZEN3600 (Malvern Instruments Ltd., Malvern, UK). The samples were diluted to a final Fe^3+^ concentration in the range of 0.1–0.3 mg/mL with water and were measured in backscattering mode at 173° at a temperature of 25 °C.

The ferrozine test was prepared by mixing 385.4 mg of ammonium acetate, 3.213 mg of ferrozine, 2.707 mg of neocuproine, and 352.24 mg of ascorbic acid, and then diluting the mixture with 1 mL of distilled water. The concentration of iron in the samples was determined as follows: 20 μL of the aqueous sample and 80 μL of concentrated hydrochloric acid were mixed in a test tube, heated for one hour at 80 °C, or kept in an ultrasonic bath for 30 min until the nanoparticles were completely dissolved. After this, the volume of the solution was brought to 10 mL with distilled water. Next, 400 μL of the obtained sample, 200 μL of distilled water, and 40 μL of ferrozine were mixed in a new test tube. The contents were then transferred to two wells of a 96-well microplate (300 μL in each), and the absorption of the solutions was measured at a wavelength of 560 nm using a Thermo Scientific Multiskan GO microplate spectrophotometer. The concentration of NPs (by Fe) was determined using a calibration curve constructed from standard solutions in the concentration range of 0.1–1 mg/mL.

#### 2.2.5. Hydrophilization of MNPs and HAEE-Cy5 Conjugation

Hydrophilization of MNPs was performed according to a previously described protocol [12]: in 10 mL of methanol CH_3_OH, 24 mg NaOH was dissolved, followed by the addition of 51 mg of DOPAC. Then, 10 mL of hydrophobic MNPs in toluene (C(Fe) = 0.5 mg/mL) were added to the prepared mixture. The mixture was first incubated for 5 h at 50 °C using a water bath under vigorous magnetic stirring and then overnight at room temperature. After cooling the mixture to room temperature, the modified nanoparticles were separated from the supernatant by centrifugation for 20 min at 6000 rpm and redispersed in 10 mL of pure deionized water. Modified nanoparticles were washed three times with pure water using centrifugal filters (Millipore Amicon Ultra-4, MWCO 30 kDa) and separated from any aggregates by passing through 0.45 and 0.22 μm syringe filters, Millex-HV, successively.

To improve the stability of the MNPs-DOPAC, additional stabilization was carried out with polyethylene glycol. For this, 2 mL MNPs-DOPAC water solution with 0.25 mg [Fe]/mL were mixed with 8 μL NHS water solution (1 mg/mL) and 12 μL EDC water solution (1 mg/mL) and incubated at room temperature for 15 min; then, 54 μL NH_2_-PEG-COOH (PEG, M_n_~3000 g/mol) solution in DI water (50 mg/mL) were added, and the resulting mixture was incubated for 12 h at room temperature. The functionalized MNPs-DOPAC-PEG were separated from the excess PEG by gel filtration using a PD-10 minicolumn with Sephadex G-25 (eluent–water), followed by filtration using 0.45 μm syringe filters Millipore.

For HAEE-Cy5 (or Cy5), conjugation was performed using the same protocol. For this, 2 mL MNPs water solution with 0.25 mg [Fe]/mL were mixed with 8 μL NHS water solution (1 mg/mL) and 12 μL EDC water solution (1 mg/mL) and incubated at room temperature for 15 min; then, 25 μL HAEE-Cy5 (10 mM) or 25 μL Cy5 (10 mM) were added, and the resulting mixture was incubated for 12 h at room temperature. The functionalized MNPs-HAEE-Cy5 and MNPs-Cy5 were separated from the excess HAEE-Cy5/Cy5 by gel filtration using a PD-10 minicolumn with Sephadex G-25 (eluent–water), followed by filtration using 0.45 μm syringe filters Millipore.

#### 2.2.6. Cell Studies

The neuroblastoma cell line SH-SY5Y was cultured in Dulbecco’s Modified Eagle Medium and Ham’s F12 (1:1) culture medium (DMEM/F12) supplemented with 10% fetal bovine serum (Gibco, Waltham, MA, USA), 2 mM L-Glutamine, 100 U/mL of penicillin, and 100 μg/mL of streptomycin. The cells were maintained at 37 °C in a humidified incubator MCO-18AC (Sanyo, Moriguchi, Japan) supplied with 5% CO_2_. After attaining 80% confluence, the cells were harvested with TrypLE and subcultured at 1:8. Cell cultures were tested for the absence of mycoplasma.

#### 2.2.7. Confocal Microscopy

The SH-SY5Y cells were seeded into a Petry dish in 1.5 mL of growth medium (500 × 10^3^ cells/dish) and cultured for 24 h. After that, the growth medium was replaced with Aβ in DMEM/F12 without FBS. The cells were incubated for 4 h, then it was twice washed using HBSS (with calcium and magnesium ions) and incubated with HAEE or MNPs-HAEE or MNPs-Cy5 in DMEM/F12 without FBS for 2 h. Finally, the cells were washed twice using HBSS (with calcium and magnesium ions). Cell imaging was performed using a Nikon Eclipse Ti2 (Nikon, Tokyo, Japan) microscope equipped with a laser scanning system (ThorLabs, Newton, NJ, USA) and Apo 25×/1.1 water immersion objective lenses. Scanning was performed using the ThorImageLS (version 2.4) software (Thorlabs, Newton, NJ, USA); Fiji 2.9.0 software was used to process the images.

## 3. Results

### 3.1. In Vitro Tests of the HAEE Effectiveness

The first step in developing a delivery system involved designing the targeting ligand. It had to contain a fluorescent label for detecting MNPs binding to Aβ on cells and an NH_2_-group for conjugation with free -COOH groups on the MNPs surface via the NHS/EDC technique. Thus, the modified HAEE (HAEE-Cy5) was used in this work. Specifically, a subsequence GGGGKK-amide was added to HAEE via a peptide bond. The first lysine residue was previously conjugated to the Cy5 fluorescent label, while the second one provided a free ε-NH_2_ group for further conjugation to the MNPs. Additionally, both N- and C-ends were modified with acetyl and amide groups correspondingly to prevent side reactions during conjugation via the NHS/EDC technique.

**Figure 1:**
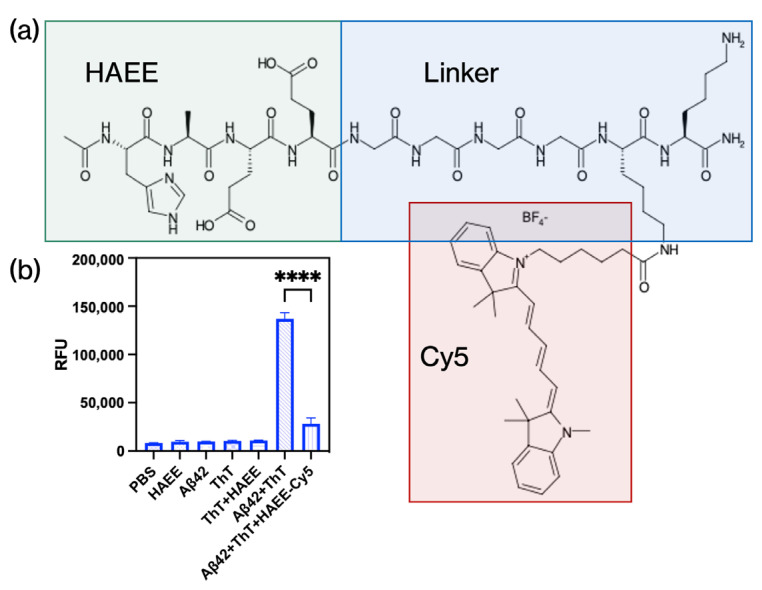
(**a**) Structural formula of HAEE-Cy5; (**b**)–ThT fluorescence assay of Aβ1–42 without and with HAEE-Cy5 after 4 h incubation, ****—*t*-test, *p* < 0.0001.

The ability of HAEE to bind and destroy aggregates of Aβ molecules has been shown previously in studies [5,13]. The modification of HAEE should not significantly affect its interaction efficiency with Aβ. It is worth noting that modification of the molecule of HAEE should not affect the efficiency of its interaction with Aβ. We used a simple ThT assay, in which ThT was shown to increase its fluorescence when added to Aβ in aggregated state, for example, when fibrils are formed (Figure 1b) [14]. When ThT is added to samples containing b-sheet-rich deposits, such as the cross-β-sheet quaternary structure of amyloid fibrils, it increases its fluorescence with excitation and emission maxima at approximately 440 and 490 nm, respectively [15]. Co-incubation of Aβ with HAEE-Cy5 significantly reduced ThT fluorescence intensity, which indicates the prevention of Aβ aggregation.

The next step was to study the interaction of HAEE-Cy5 and Aβ with the cells. In this work, we have chosen the SH-SY5Y cells for modeling AD in vitro. Although the use of cancer cells may not be preferred when comparing with neurons and astrocytes, these cells are often used for in vitro models of neuronal function and differentiation because of their morphological neuroblast-like form [16].

**Figure 2:**
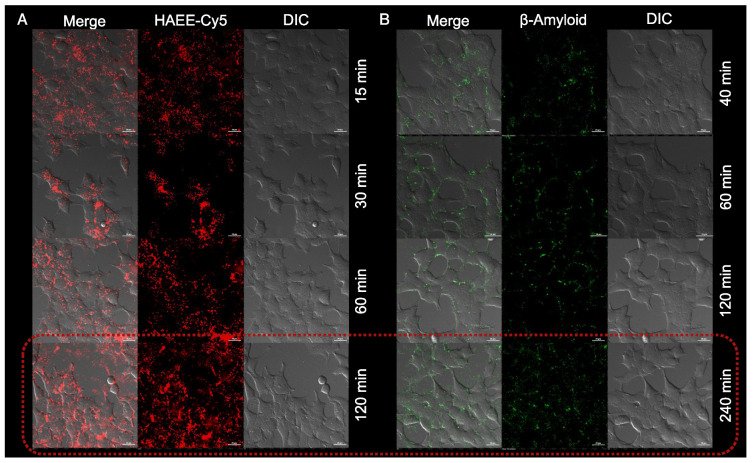
(**A**)—HAEE-Cy5 interaction with SH-SY5Y cells for 120 min at an HAEE concentration 10 μM; (**B**)—Aβ1-40 interaction with SH-SY5Y cells for 240 min at an Aβ1-40 concentration = 1 μM. Laser scanning confocal microscopy, scale bar 20 μm.

**Figure 3:**
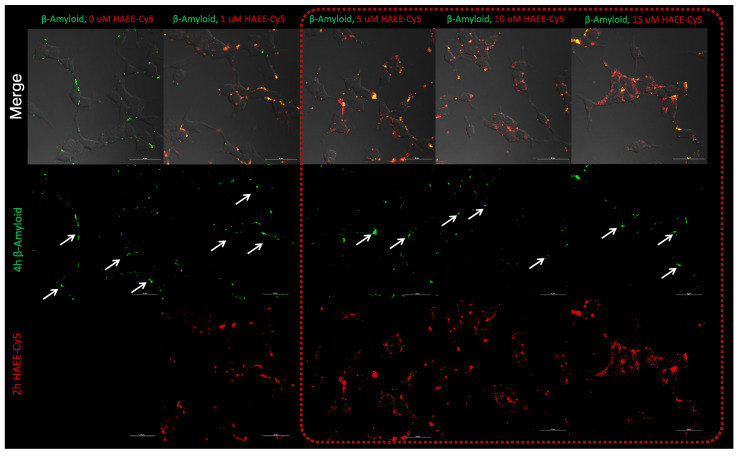
The inhibitory influence of different concentrations of HAEE-Cy5 on Aβ1-40 aggregation on SH-SY5Y cells. Laser scanning confocal microscopy, scale bar 30 μm. White arrows are indicating Aβ1-40 aggregates.

As expected, both HAEE-Cy5 and Aβ were detected mostly within the cell membrane (Figure 2) and were only slightly internalized into the cells. The maximum accumulation of Aβ and HAEE-Cy5 was reached after 240 and 120 min of incubation, respectively. Also, different concentrations of HAEE-Cy5 were tested. We have observed that HAEE concentrations higher than 5 μM lead to a reduction in the size of amyloid plaques after 120 min incubation (Figure 3). This nicely corresponds with previously published results, where significant effects on Aβ disaggregation were shown for concentrations of HAEE equal to 10 μM [5].

### 3.2. Synthesis and Characterization of MNPs

**Figure 4:**
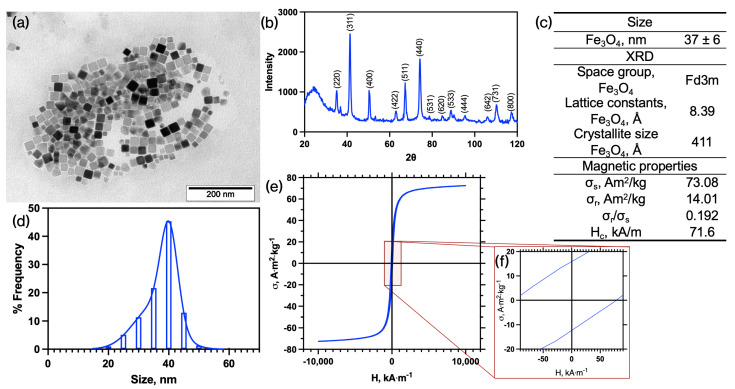
(**a**) TEM image of MNPs; (**b**) XRD patterns of the MNPs powder; (**c**) results of TEM, XRD, and magnetic loop analysis of the MNPs; (**d**) frequency distribution of the MNPs (bimodal Gaussian distribution); (**e**) magnetic hysteresis loop of the MNPs from PPPMS measurements; (**f**) area of the magnetic hysteresis loop of the MNPs from PPPMS measurements. σ_s_—specific saturation magnetization; σ_r_—specific residual saturation; H_c_—Coercive force.

MNPs were prepared by the thermal decomposition of Fe(acac)_3_ in a high-boiling solvent in the presence of stabilizers. Similar MNPs have been previously reported for various applications [10,11]. In this study, we aimed to obtain stable nanoparticles with a sufficiently high saturation magnetization to improve contrast in MR images. We obtained cubic nanoparticles with a size of approximately 40 nm in size, consisting of magnetite (Figure 4). Based on the broadening of diffraction lines, the calculated crystallite size was 41 ± 5 nm. The calculated value of microstrains (ε = 0.01%) indicates the nanoscale nature of the particles in the sample. The specific saturation magnetization was 73 Am^2^/kg, close to that of bulk magnetite, suggesting high T₂ relaxivity values for enhanced MRI contrast [17], sufficient for detection of even small targets such as amyloid-damaged brain areas.

### 3.3. Coating of MNPs

The MNPs obtained via thermal decomposition have a hydrophobic shell composed of OA and OAm, making them unstable in aqueous solutions. At the same time, for effective delivery of the MNPs to the brain, they must maintain colloidal stability in water and aqueous solutions with pH and ionic strengths mimicking physiological fluids.

**Figure 5:**
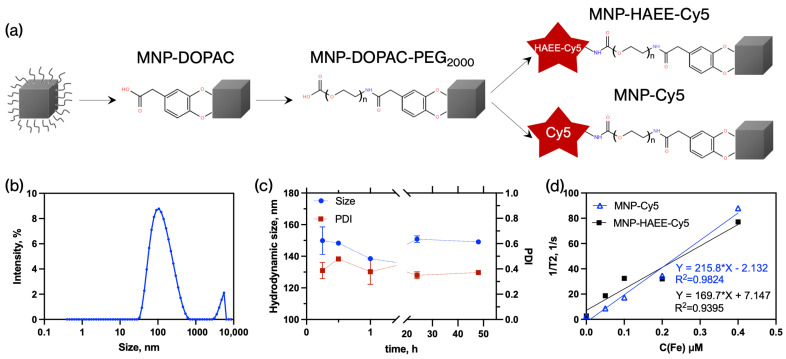
(**a**) Scheme of DOPAC and PEG coating with MNPs and covalent conjugation with Cy5 and with HAEE-Cy5; (**b**) DLS measurement of MNPs-DOPAC-PEG size by intensity distribution; (**c**) Hydrodynamic stability of MNPs-HAEE-Cy5 in culture medium supplemented with 10% FBS; (**d**) dependence of the 1/T2 relaxation parameter on the iron concentration in MNPs-Cy5 and MNPs-HAEE-Cy5 samples.

To transfer these NPs into water, DOPAC molecules can be used as previously demonstrated [12]. As a result, stable in distilled water MNP dispersions are formed. Bifunctional NH_2_-PEG-COOH was used for additional stabilization (Figure 5a). It is also necessary to take into account that one of the objectives of this work is to develop a system that can potentially increase the circulation time of HAEE in the bloodstream. The primary factors that reduce the circulation time are the capture of the reticuloendothelial system (RES) organs, such as the liver and spleen. PEG is a well-known approach to avoid these undesirable effects.

Through sequential reactions, MNPs with a hydrodynamic size of 119 ± 3 nm and a polydispersity index of 0.348 ± 0.003 were produced. The carboxyl group on the PEG allows for further functionalization with HAEE-Cy5 and Cy5. After conjugation of HAEE-Cy5 with the nanoparticles, HAEE-Cy5 was equal to 67 nmol per 1 mg Fe. Iron and Cy5 concentrations were measured by ferrozine assay and Cy5 absorbance (Supplementary Figure S2). TEM images of MNP-HAEE-Cy5 indicate the appearance of a thin layer around the iron oxide core, probably consisting of DOPAC, PEG, and HAEE-Cy5 molecules (Supplementary Figure S3). Additionally, MNP-HAEE-Cy5 retained hydrodynamic stability for at least 48 h in a growth medium with FBS (Figure 5c). As a control sample, MNP without any targeting ligand (MNP-Cy5) was synthesized.

Measurements of T2 relaxation of MNP-HAEE-Cy5 have shown R2 values equal to 216 and 170 mM^−1^s^−1^ for MNPs-Cy5 and MNPs-HAEE-Cy5, respectively (Figure 5d).

**Figure 6:**
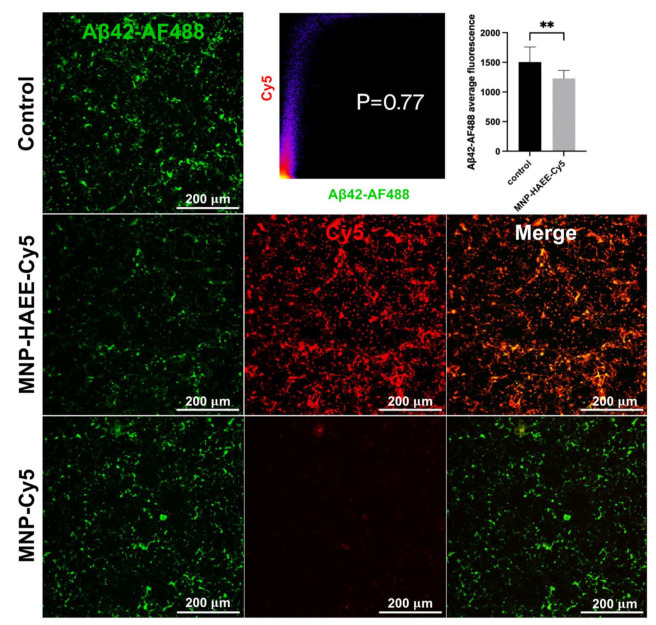
MNPs-HAEE-Cy5 and MNPs-Cy5 interaction with SH-SY5Y cells pre-incubated Aβ1-42-AF488, laser scanning confocal microscopy, scale bar 200 μm; the correlogram between Cy5 and Aβ1-42-AF488 for MNP-HAEE-Cy5-images; comparing of fluorescence intensity of Aβ1–42 without and with MNP-HAEE-Cy5 after 4 h incubation, **—*t*-test, *p* < 0.01.

Finally, the use of MNPs-HAEE-Cy5 demonstrated a fairly high level of binding to labeled Aβ (Figure 6). At the same time, MNPs-Cy5 showed almost no interaction with cells preincubated with Aβ. The value of the Pearson coefficient is significantly lower than 1, which is partly explained by the extremely high fluorescence signal from the MNP-HAEE-Cy5. Additionally, it should be noted that the concentration of beta-amyloid fibrils decreases, as indicated directly by a significant reduction in fluorescence compared to the control (Figure 6). Also, MTS tests have shown no significant effects on cell viability after incubation with SH-SY5Y cells (Supplementary Figure S4). These results indicate the high specificity of the developed nanoparticles, suggesting their potential for further in vivo testing.

## 4. Discussion

Currently, there are not many ways to diagnose Alzheimer’s disease (AD). In clinical practice, for example, spinal fluid analysis is used to detect the presence of Aβ and tau proteins [18]. However, the use of instrumental analysis methods, such as MRI, especially in the early stages of the disease, is challenging for several reasons: (1) the affected areas of the brain are quite small and difficult to distinguish from healthy ones and (2) currently, there are no approved probes capable of increasing the contrast of affected brain areas using MRI.

In clinical practice, single-photon emission computed tomography (SPECT) with exametazime is used, which allows the diagnosis and differentiation of dementia based on the distribution patterns of a radiopharmaceutical in the brain [19,20]. This method helps to distinguish between frontotemporal degeneration and AD [21], but it cannot differentiate AD from Lewy body dementia due to the great similarity in patterns [22]. Another method to diagnose AD is positron emission tomography (PET) with the use of ^11^C Pittsburgh Compound B [23]. However, even with the results obtained, it is not possible to accurately determine the type of dementia, and these methods are quite expensive and require the use of radioactive markers, while MRI diagnostics could be much cheaper.

MNPs have long been used in biomedicine for cancer diagnosis with MRI [14], drug delivery [24], magnetomechanics [25,26], and hyperthermia [27]. The type of particles chosen depends on the specific application.

The use of magnetic particles for the diagnosis of AD has been poorly explored; in previous studies, particles with relatively low magnetization (around 50 emu/g) were used, and the targeting molecules had far from optimal specificity [8,27]. In our case, we needed to develop particles with good MRI contrast that could eventually be applied in in vivo diagnostics. It has been shown that particles in the form of cubes and octopods provide the best MRI contrast compared to spherical particles. This is due to the fact that magnetic flux spreads through the corners of the cube in a pattern resembling flower petals, which in turn leads to more complex induced local magnetic fields and an increased water relaxation rate [28]. The DOPAC-PEG coating was chosen not only because it provides good colloidal stability and -COOH group for conjugation with HAEE-Cy5, but also because of its low unspecific uptake by SH-SY5Y and RAW 264.7 cells in vitro [29]. Also, use of HAEE as a targeting molecule significantly increases binding efficiency to Aβ on the cell surface. Additionally, it should be noted that this type of nanoparticle will not only have a reduced uptake by RES cells but also by brain cells. This is an important consideration in this work, as the target protein Aβ is also located in the extracellular space, and the uptake of diagnostic nanoparticles is highly undesirable. Finally, a cubic-shaped iron oxide magnetic core results in high magnetization and T2 relaxivity values, allowing further applications as an MRI contrast agent. DOPAC-PEG coating provides high colloidal stability and reduces unspecific uptake, whereas conjugation with HAEE allows specific interactions with Aβ amyloid on the SH-SY5Y surface.

## 5. Conclusions

In this study, the HAEE tetrapeptide was modified by adding a subsequence of GGGGKK-amide, as well as a fluorescent label, Cy5. These modifications allowed the peptide to be covalently conjugated to MNPs while retaining its ability to interact with Aβ and break down Aβ fibrils. HAEE-Cy5 conjugated to MNPs retained their Aβ targeting interactions and exhibited a high loading capacity, making them promising for future applications in AD diagnosis by MRI.

## Data Availability

The raw data supporting the conclusions of this article will be made available by the authors on request.

## Supplementary Materials

The following supporting information can be downloaded at: https://www.mdpi.com/article/10.3390/pharmaceutics16111395/s1, Figure S1. (a) ThT fluorescence spectra after 2 h incubation; (b) Absorption spectrum of HAEE-Cy5; (c) Fluorescence spectrum of HAEE-Cy5. Figure S2. (a) Calibration curve; (b) MNPs-DOPAC-PEG and MNPs-HAEE-Cy5 absorbance in water solution; (c) MNPs-HAEE-Cy5 absorbance after subtraction MNPs-DOPAC-PEG absorbance. Figure S3. TEM-image of MNPs-HAEE-Cy5. Figure S3. TEM-image of MNPs-HAEE-Cy5. Figure S4. Cytotoxicity of MNPs-HAEE-Cy5.
