# Supplementary material for: Targeted Magnetic Nanoparticles for Beta-Amyloid Detection
Source: Pharmaceutics. 2024 Oct 29;16(11):1395. doi: 10.3390/pharmaceutics16111395 (PMC11597426; doi:10.3390/pharmaceutics16111395)
Supplement: Supplementary file 1 [file pharmaceutics-16-01395-s001.zip › pharmaceutics-3253361-supplementary.pdf]

# Supplementary Materials

# Targeted Magnetic Nanoparticles for Beta-Amyloid Detection

**Nelly S. Chmelyuk, Aleksey A. Nikitin, Veronika V. Vadekhina, Vladimir A. Mitkevich and Maxim A. Abakumov**

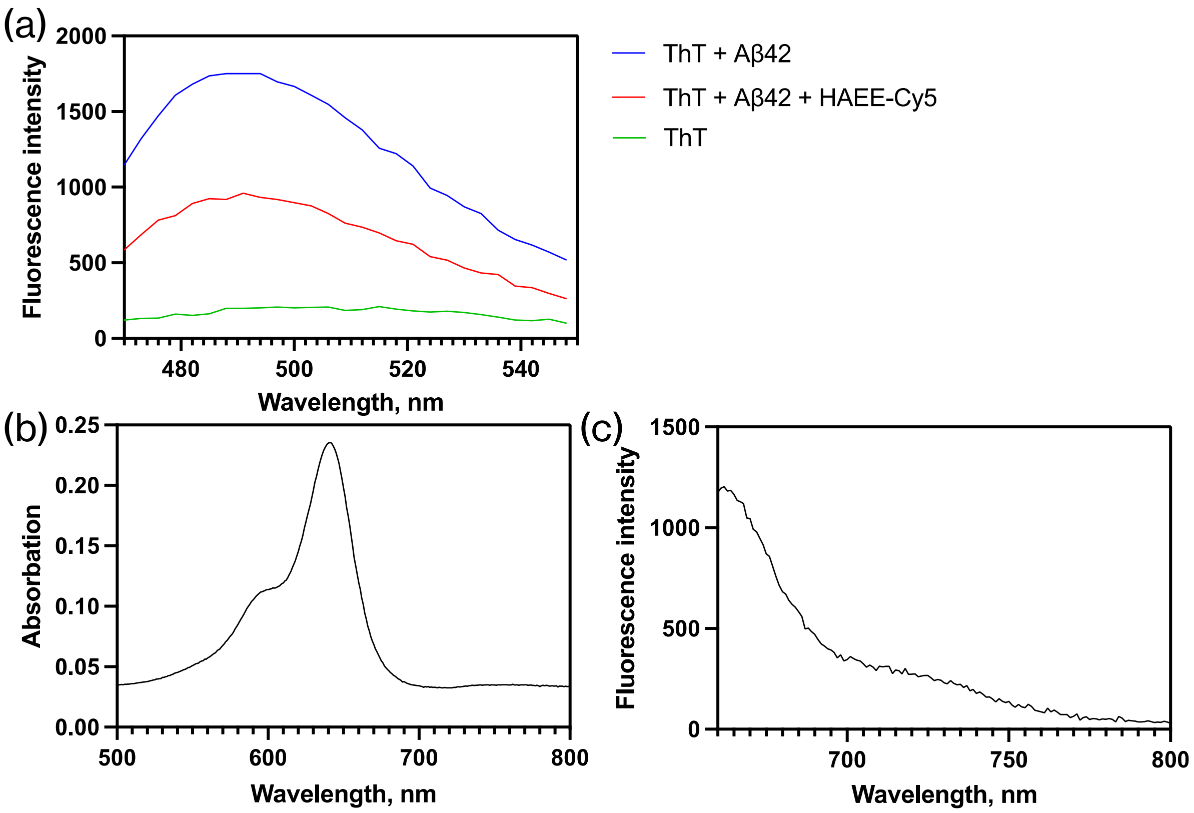


**Figure S1.** (**a**) ThT fluorescence spectra after 2h incubation; (**b**) Absorption spectrum of HAEE-Cy5; (**c**) Fluorescence spectrum of HAEE-Cy5.

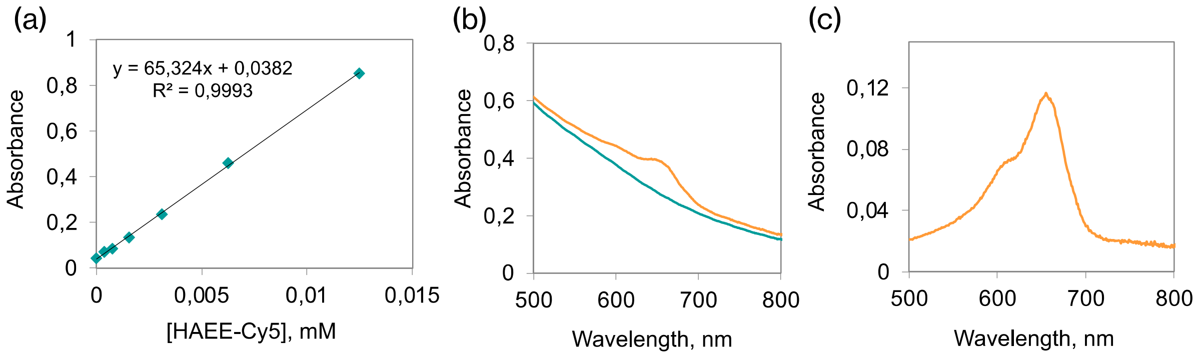


**Figure S2.** (**a**) Calibration curve; (**b**) MNPs-DOPAC-PEG and MNPs-HAEE-Cy5 absorbance in water solution; (**c**) MNPs-HAEE-Cy5 absorbance after subtraction MNPs-DOPAC-PEG absorbance.

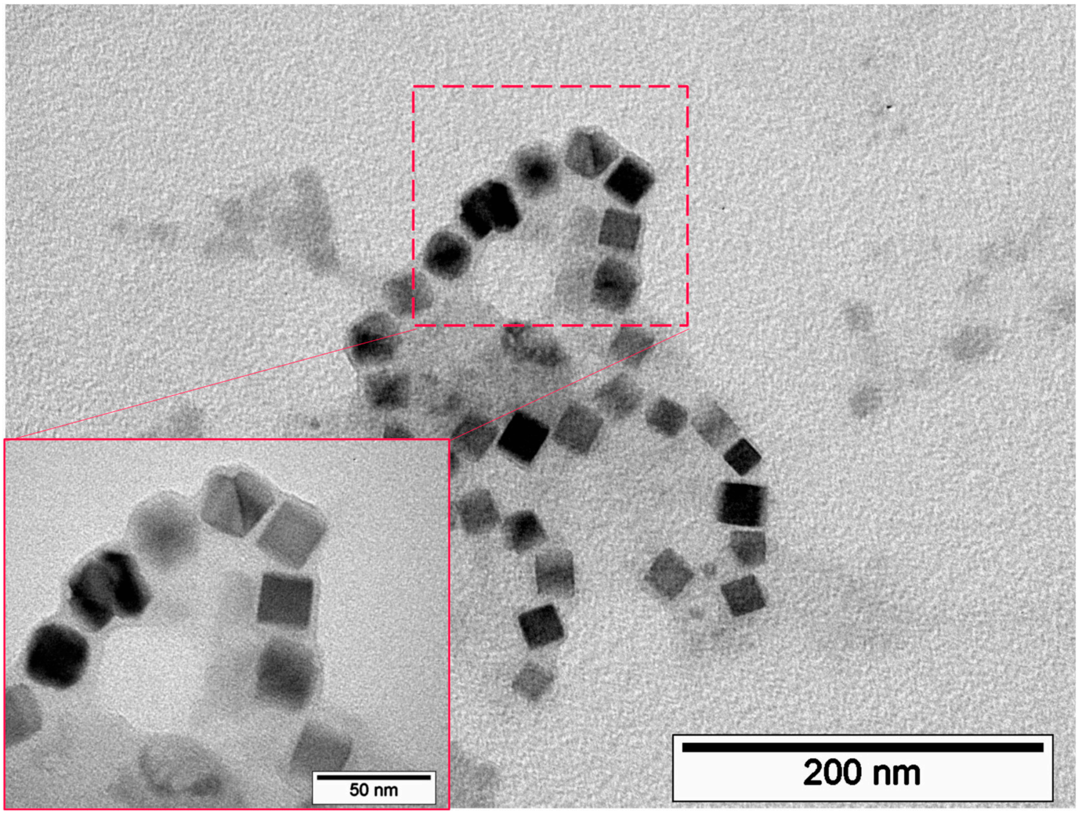


**Figure S3.** TEM-image of MNPs-HAEE-Cy5.

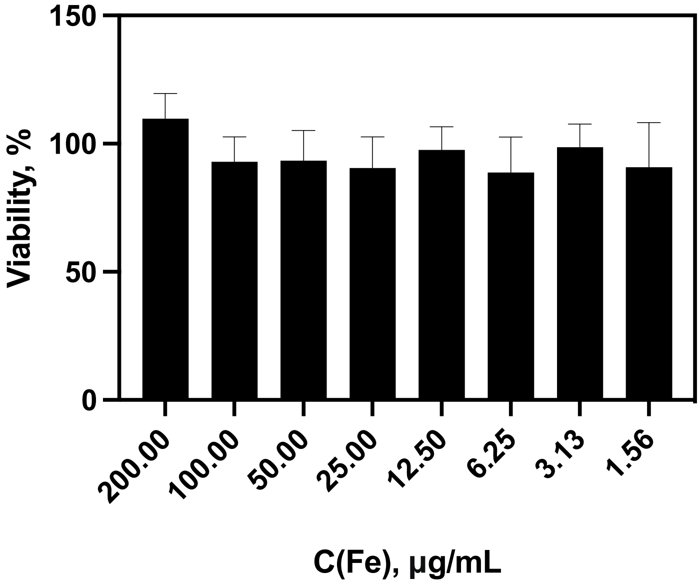


**Figure S4.** Cytotoxicity of MNPs-HAEE-Cy5.
